# Supplementary material for: Impactor material records the ancient lunar magnetic field in antipodal anomalies
Source: Nat Commun. 2021 Nov 11;12:6543. doi: 10.1038/s41467-021-26860-1 (PMC8586259; doi:10.1038/s41467-021-26860-1)
Supplement: Supplementary file 1 — Supplementary Information [file 41467_2021_26860_MOESM1_ESM.pdf]

Supplementary Information

Impactor material records the ancient lunar magnetic field in antipodal anomalies

Authors

S. Wakita[1,2]*, B. C. Johnson[1,3], I. Garrick-Bethell[4, 5], M. R. Kelley[4], R. E. Maxwell[4], T. M. Davison[6].

Affiliations

[1]Department of Earth, Atmospheric, and Planetary Sciences, Purdue University, West Lafayette, IN 47907, USA.

[2]Department of Earth, Atmospheric and Planetary Sciences, Massachusetts Institute of Technology, Cambridge, MA 02139, USA.

[3]Department of Physics and Astronomy, Purdue University, West Lafayette, IN 47907, USA.

[4]Department of Earth and Planetary Sciences, University of California, Santa Cruz, CA 05064, USA.

[5]School of Space Research, Kyung Hee University, Yongin, Gyeonggi, 446-701, Korea.

[6]Department of Earth Science and Engineering, Imperial College London, London SW7 2AZ, UK.

Supplementary information

Supplementary Note 1. Data sources for magnetic field and iron maps.

Supplementary Note 2. Impact basin size and shape.

Supplementary Note 3. Ejecta thickness calculations.

Supplementary Note 4. Impactor material around the crater.

Supplementary Note 5. Effect of rotation.

Supplementary Figure 1. Modeling of the magnetic sources at the strongest anomalies at the Crisium antipode.

Supplementary Figure 2. Wide field view of the Crisium antipode magnetic anomaly.

Supplementary Figure 3. Lunar geologic map draped over greyscale topography.

Supplementary Figure 4. Crater floor elevation at Houzeau and two craters of similar diameter.

Supplementary Figure 5. Spatial distribution of ejecta landing on the antipodal hemisphere.

Supplementary Figure 6. Ejecta distribution on the antipodal hemisphere with color representing their flight time in hours.

Supplementary Figure 7. Peak pressure of antipodal ejecta sourced from the impactor as a function of the provenance depth in the impactor of 12 km/s.

Supplementary Figure 8. Peak pressure of antipodal ejecta sourced from the impactor as a function of their flight time.

Supplementary Figure 9. Thickness of ejecta around the antipode.

Supplementary Figure 10. Time series of basin formation.

Supplementary Figure 11. Ejecta distribution around the crater.

Supplementary Table 1. Antipodal thickness and its origin.

Supplementary Table 2. iSALE input parameters.

Supplementary References

Supplementary Notes

Data sources for magnetic field and iron maps

The data for Fig. 1a comes from the spherical harmonic model of Tsunakawa et al. 2015 [1], evaluated at 20 km altitude. Data for Supplementary Fig. 1a comes from the same model at the lunar surface. The model is derived from a combination of Lunar Prospector and Kaguya measurements at ~20 km altitude, so we prefer the model output at 20 km altitude to assess correlations with geology, but the surface field values help to show the overall spatial extent of the anomaly.

Iron data for Supplementary Fig. 2b come from gamma ray measurements from the Lunar Prospector mission [2].

Impact basin size and shape

We can estimate the impactor size assuming the impact conditions (such as impact velocity, angle, and target properties) using scaling laws. Based on the scaling law [3], the Crisium basin (1076 km) can be formed by the impactor of 92 km in diameter with 45º at 12 km/s. Since another scaling law for a different target property [4] also indicates similar results at the same impact velocity and angle (main text), our setting of 100 km is valid for the formation of the Crisium basin (see below). Note that high velocity impact makes a larger basin; e.g., the impactor of 100-200 km at 45º with 18 km/s could form the Imbrium size basin [5]. Since the shape of basin produced by an impact angle larger than 40º is almost circular [6], it is impossible to estimate the impact angle only by its shape. A 30º impact represents intermediate shape between circular and elliptical. Thus, oblique impacts of 45º and 30º may form nearly circular basins and the antipodal magnetic anomalies at the same time.

Ejecta thickness calculations

When we derive the ejecta thickness on the antipodal hemisphere (Fig. 4), we sum the tracer particles and consider their total volume in a surface area 1° in diameter (equal to 30 km assuming a lunar radius of 1737.4 km). Since the ejecta over the antipode always passes it (Supplementary Fig. 8a) and tends to gather at the antipode, the choice of area governs the thickness right at the antipode. The number of ejecta right at the antipode does not change, thus as the area shrinks the ejecta thickness becomes larger. The thickness of 45° impact at 12km/s is 350 m, 720 m, 1450 m, and 2960 m for the surface area of 4°, 2°, 1°, and 0.5° in diameter, respectively (Supplementary Fig. 8b). Our choice of 1° areas for our averaging is motivated by observed surface area of lunar swirls (Fig. 1 box s, and Supplementary Fig. 1) and the fact that 1° is the smallest area that produces smooth and continuous variations in thickness around the antipode. Although the ejecta thickness changes by the choice of area, our estimates of the ejecta thickness may be conservative and lower limits. This is because secondary craters are distributed heterogeneously [7]. We can expect that the ejecta would also have a heterogeneous distribution (Fig. 4). If we underestimate the ejecta thickness, the field strength is overestimated.

Impactor material around the crater

Lunar magnetic anomalies can be found not only antipodal to the basin, but also within and around the basin [8,9,10]. To explore location of ejecta deposits around the basin post-impact, we perform another simulation. Since our standard runs with 1 km resolution are computationally expensive to track the entire crater formation process, we use a lower resolution (10 km) with a suitable temperature profile [11]. Supplementary Figure 10 shows the snapshots of crater formation by a 100-km-diameter impactor with 45° at 12 km/s. The crater reaches its maximum excavation volume (i.e., transient crater) at ~ 300 s after the impact (Supplementary Figure 10b). The diameter of the transient crater is about 400 km, which is consistent with the 389 km transient

crater for Crisium found by Miljković et al. [4]. After the formation of a large central uplift (Supplementary Figure 10c), the crater settles down to its final state (Supplementary Figure 10d). Although it is hard to recognize the topographic expression with a 10 km resolution, the diameter of the final crater would be 900 - 1100 km. This is consistent with our estimates of ~ 1000 km (see main text). We confirm that the impactor material is distributed within the final crater (Supplementary Figure 11). Much of the impactor material experiences high pressures and temperature indicating the material can become magnetized as it cools. Note that such ejecta originate from various locations in the impactor (see the size of symbols). The impactor material is preferentially distributed downrange as shown in previous work [12]. We also find even in this low resolution simulation single tracer particle for the impactor is located within 3° of the antipode; its volume of ~ 1000 km^3 is comparable to ~ 1400 km^3 by the same impact condition but with 1 km resolution. As we observe the magnetic anomalies within the Crisium basin [10] and its antipode [8,9], our results suggest that impactor material from an oblique impact can reasonably produce magnetic anomalies within the basin and at its antipode.

Effect of rotation

Considering the ancient Moon at 4.0 Gyr ago which rotated two times faster than the current Moon [13], ejecta is displaced westward by ~1.1° during each hour of flight time. Thus antipodal ejecta may be offset by several degrees from our calculated locations (Fig. 3). If the impact antipode is on the equator each degree corresponds to 30 km of westward displacement. For an antipode at pole, however, the displacement vanishes. Note that we take the antipode from the impact site, which is the origin in the simulation. The impact site of the simulations and the observed lunar basin center is the same only for the vertical impact. As the impact site of simulated oblique impact might be different from the observed lunar basin center, the antipode also does and may have a displacement of a few degrees. A complete consideration of the effect

of rotation would require knowledge of the properties of the impactor (impact site and direction) and the lunar rotation properties (period and axis) at the time of lunar basin formation. Such properties, especially impact direction, are uncertain, meaning a wide parameter space must be explored, which is too wide to perform presently.

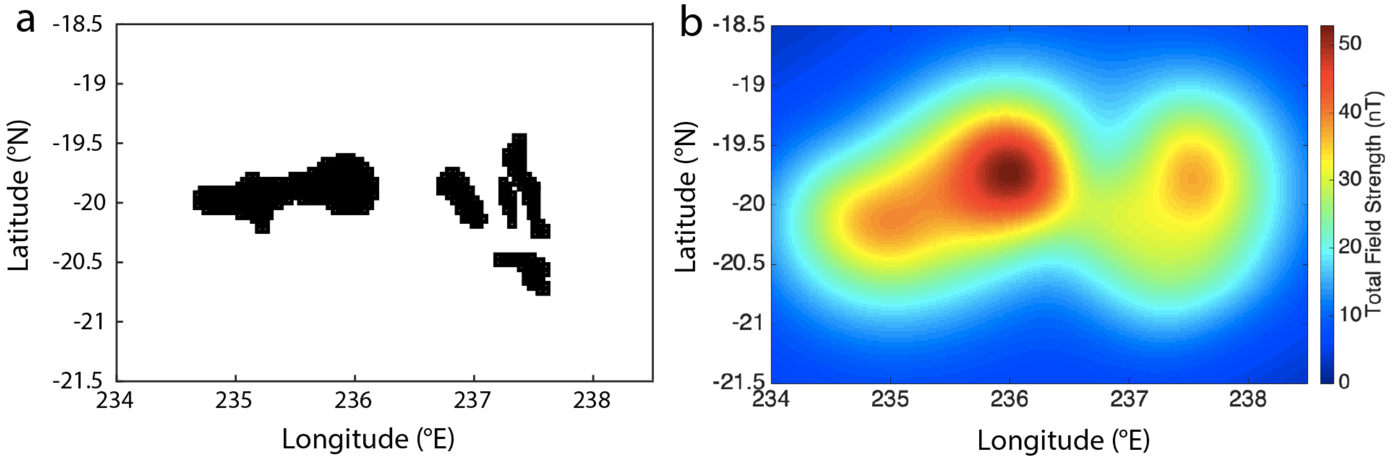

Supplementary Figure 1. Modeling of the magnetic sources at the strongest anomalies at the Crisium antipode.

(a) Two dimensional sheets of dipoles used to model the magnetization of the five swirls in the white box s shown in Fig. 1 in the main text. (b) Total magnetic field from the swirl model in panel a, evaluated 20 km above the source plane. The magnitude (~50 nT peak) and general shape of the model field are similar to observations (compare with Fig. 1a in the main text).

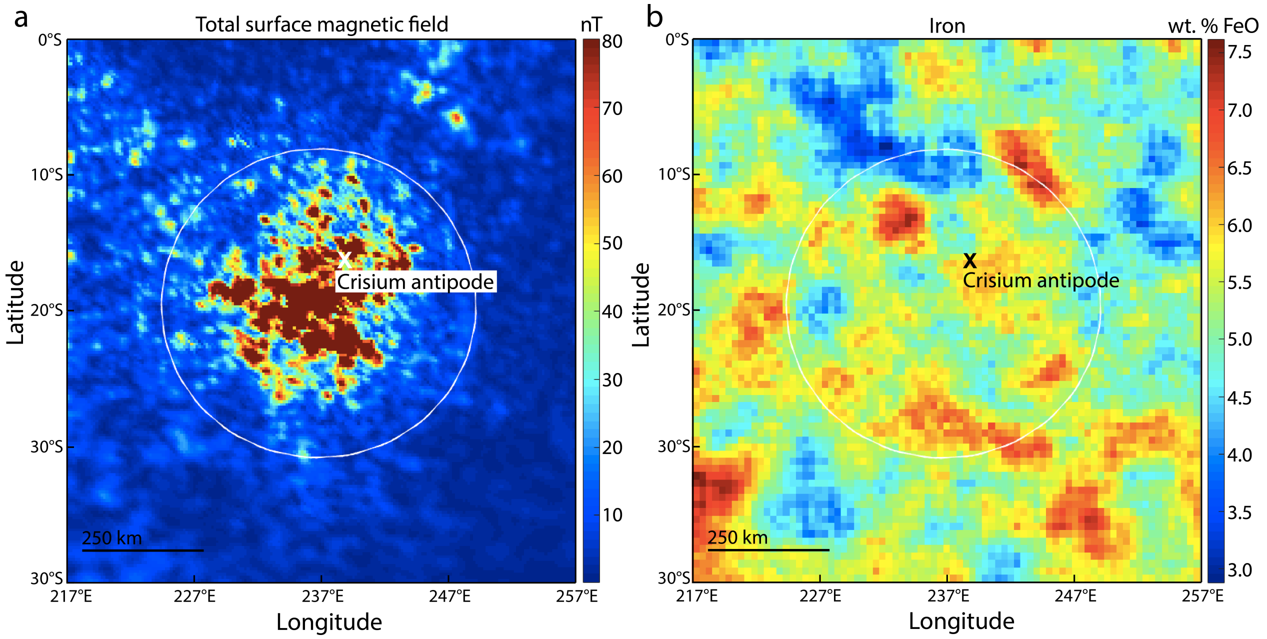

Supplementary Figure 2. Wide field view of the Crisium antipode magnetic anomaly.

(a) Surface magnetic field from Tsunakawa et al. 2015 [1]. The peak field in this region is 672 nT but the map was saturated at 80 nT (dark red values) to illustrate the wide extent and coherence of the anomalous magnetization. White circle is a small circle centered at (20º S, 237º E), with diameter of 700 km. (b) Same as panel a but for gamma-ray derived iron (Lawrence et al. 2002 [2]). Both panels are Miller projections.

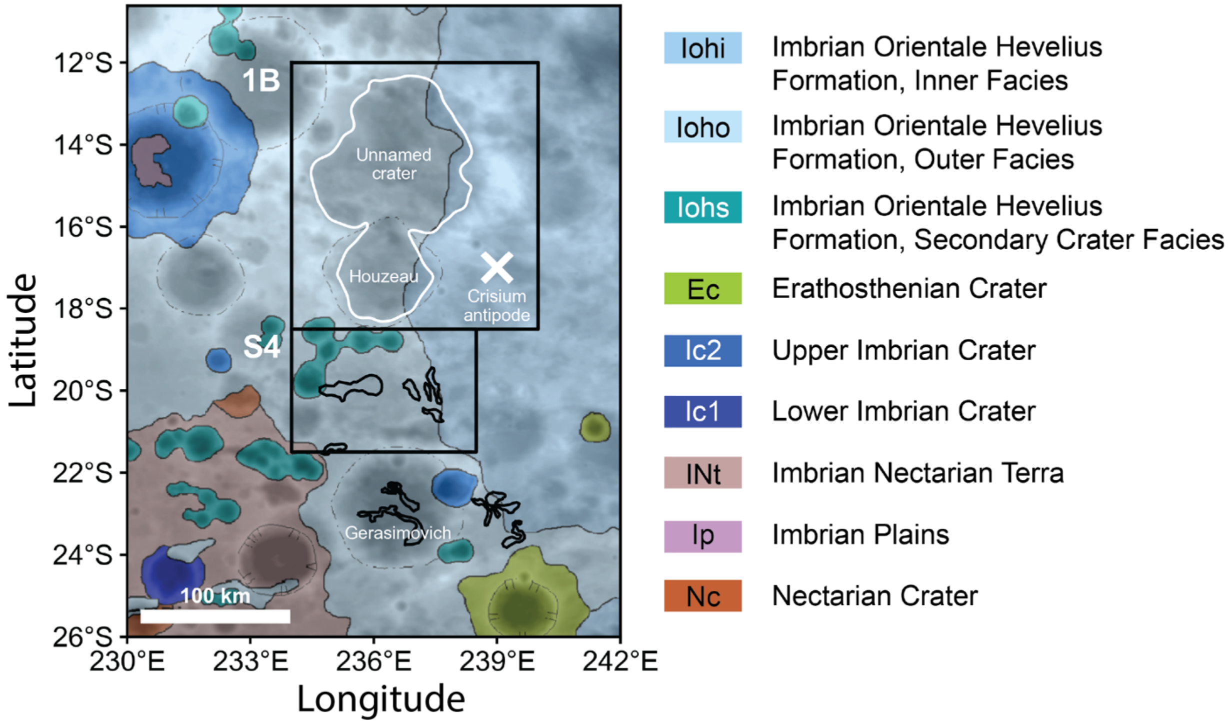

Supplementary Figure 3. Lunar geologic map draped over greyscale topography.

Lunar geologic map draped over greyscale topography, in the vicinity of the Crisium antipode and associated magnetic anomalies (compare with Fig. 1 in the main text and Supplementary Fig. 2). The magnetic anomalies are almost entirely within the outermost Orientale ejecta deposit, mapped as unit Ioho (western portion of the figure). The greyscale topography and map units are a composite image generated by https://quickmap.lroc.asu.edu. Geologic map is from Parker et al. [14] and topography data are from Smith et al. 2010 [15]. Unit definitions relevant to interpretation of figure: Iohi – Curvilinear to swirly ridges and troughs mostly radial and subradial to Orientale basin. Interpretation: Continuous ejecta blanket emplaced during outward flow of hot, turbulent, mobile materials. Ioho – Swirly, lineated, hummocky and smooth materials forming a discontinuous and irregular boundary. Interpretation: Thinning distal margins of Orientale basin ejecta.

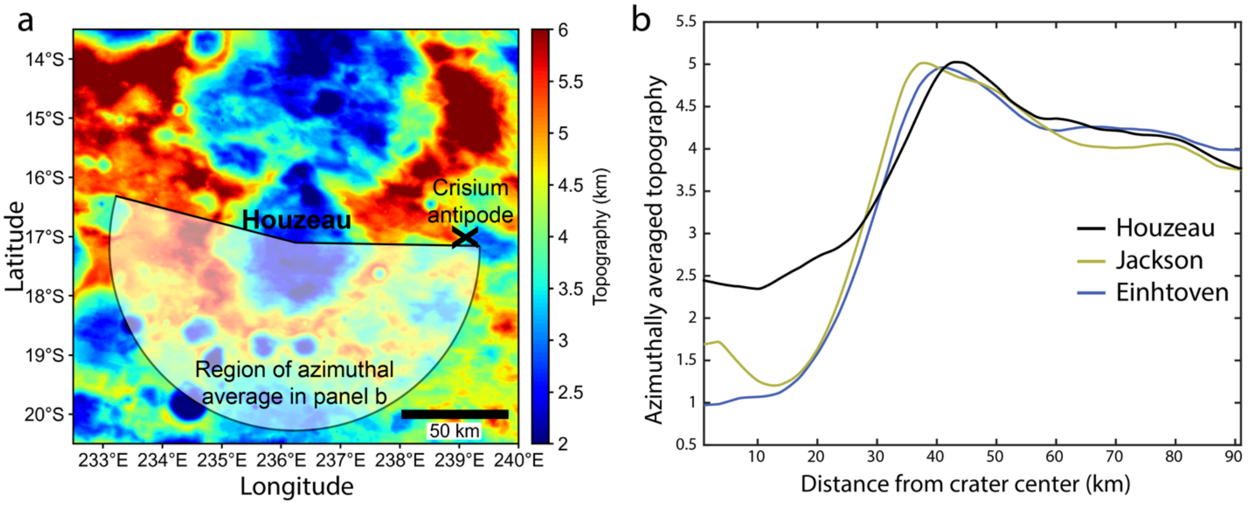

Supplementary Figure 4. Crater floor elevation at Houzeau and two craters of similar diameter.

(a) Topography map in the vicinity of the Crisium antipode (compare with Fig. 1b; data from Smith et al. 2010 [15]). The highlighted white sector shows the region used to calculate the azimuthal average of Houzeau crater's topography. This region avoids topography associated with the unnamed crater in the north. Displayed topography data are saturated at 2 and 6 km for clarity. (b) Azimuthally averaged of topography for Houzeau, and the similarly sized Jackson and Einthoven craters. Unlike Houzeau, the topography of Jackson and Einthoven craters is calculated from a full 360° of azimuth, since there are no nearby overlapping geologic features at these craters. Note that Jackson has a central peak, while Einthoven does not.

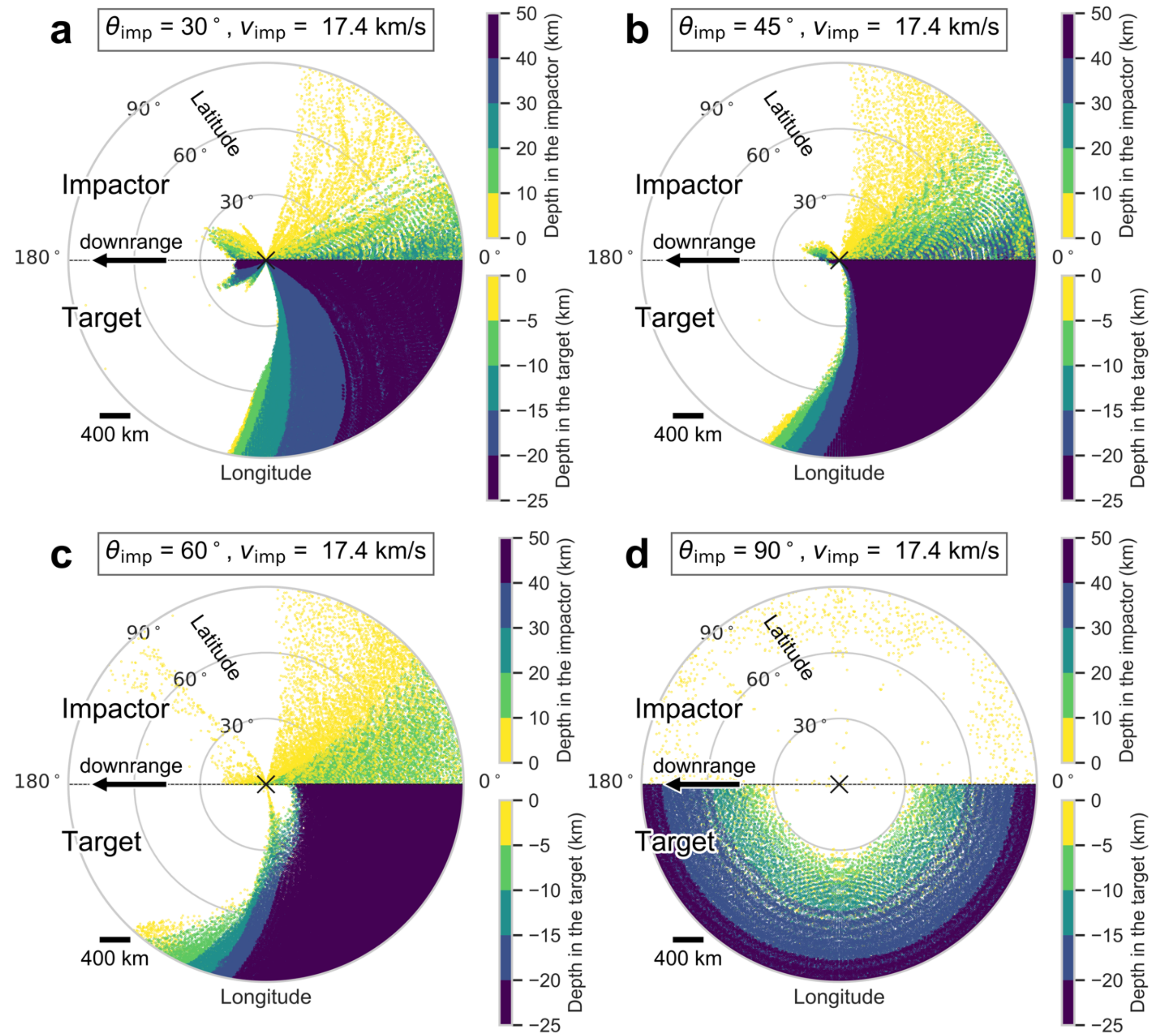

Supplementary Figure 5. Spatial distribution of ejecta landing on the antipodal hemisphere.

Same as Fig. 2 in the main text, but for the impact scenarios of 17.4 km/s.

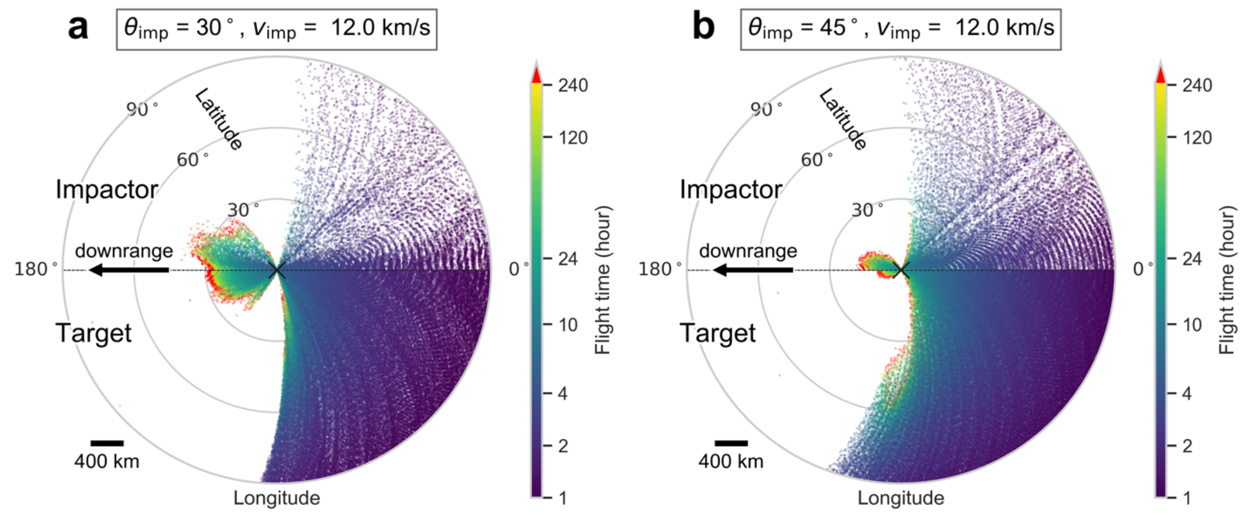

Supplementary Figure 6. Ejecta distribution on the antipodal hemisphere with color representing their flight time in hours.

Each panel shows ejecta from (a) 30º and (b) 45º impacts with 12 km/s, respectively. Top halves of each panel show ejecta originated from the impactor and bottom are ejecta originated from the target. To illustrate the ejecta with short flight times, we plot them on top. Note that in reality, ejecta with shorter flight times will be located underneath ejecta with longer flight times.

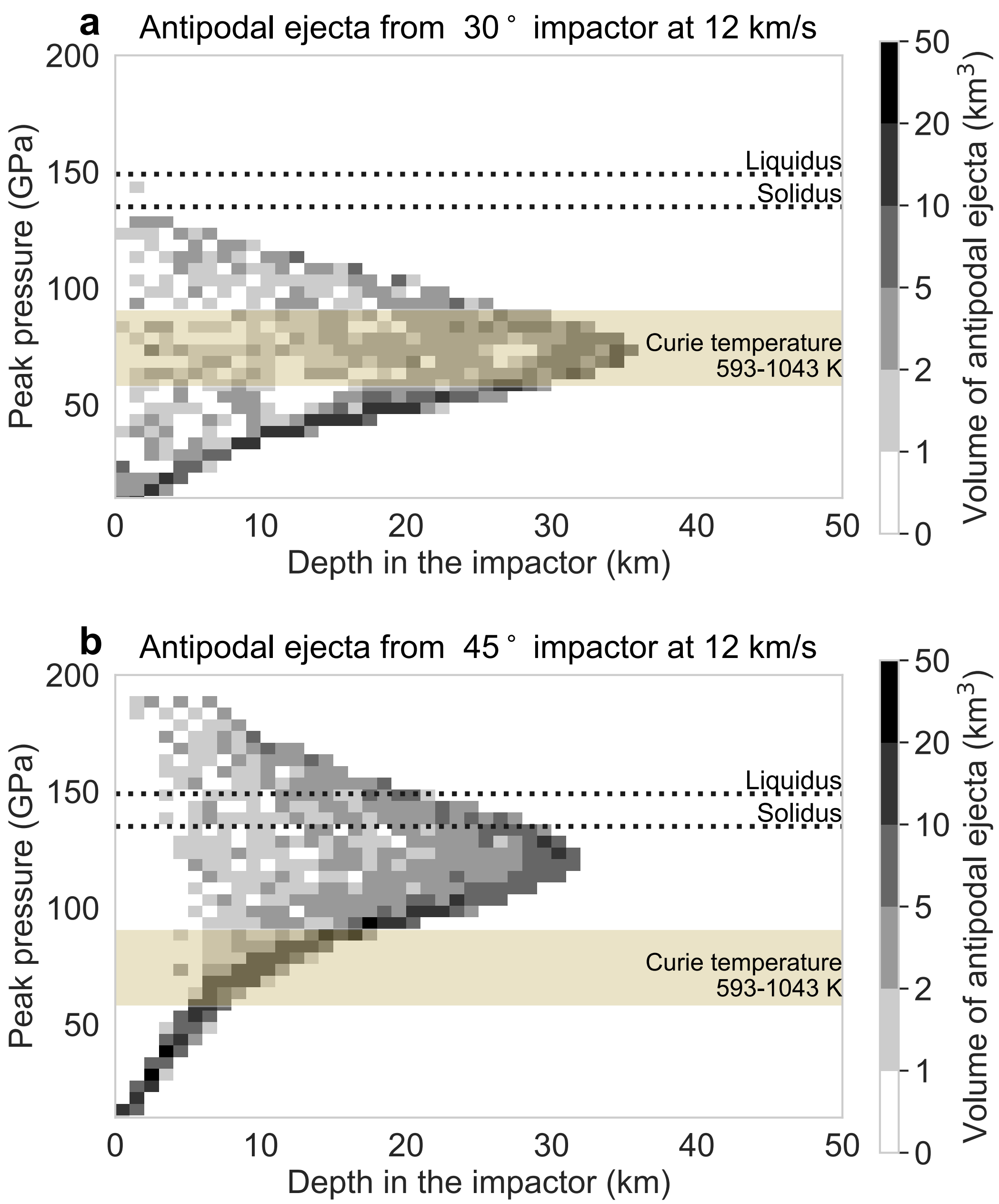

Supplementary Figure 7.

Peak pressure of antipodal ejecta sourced from the impactor as a function of the provenance depth in the impactor of 12 km/s. Each panel represents different impact angles (a) 30º and (b) 45º, respectively.

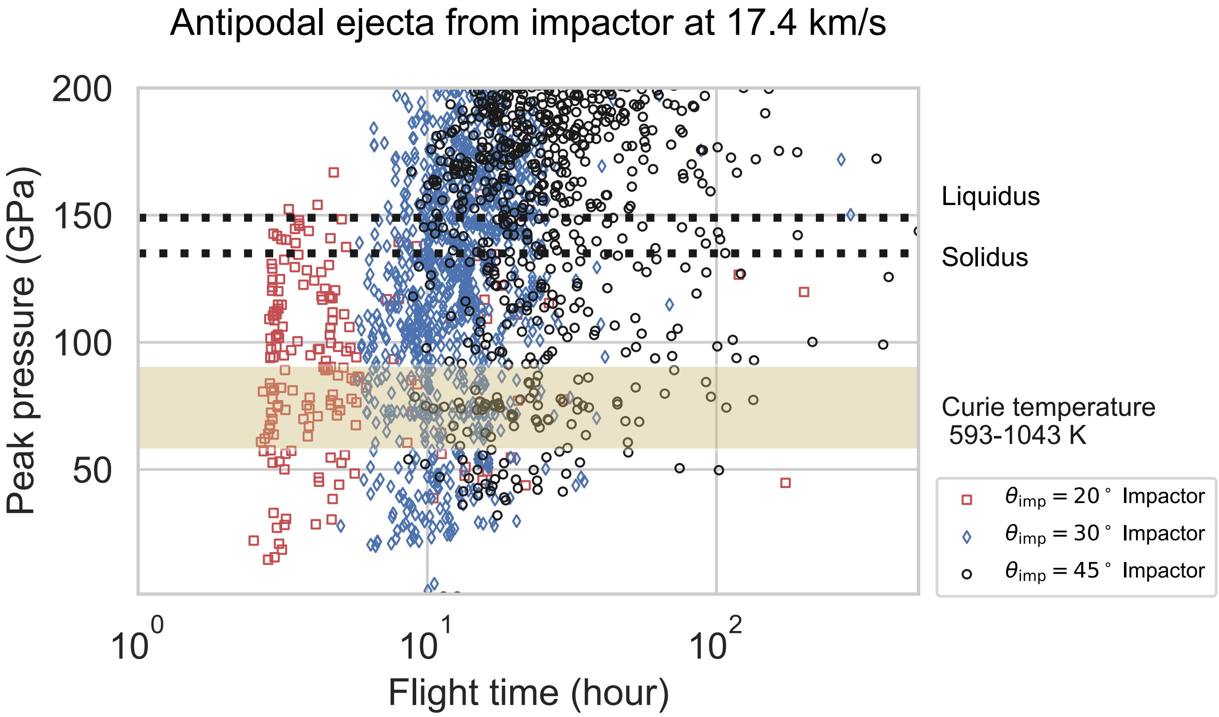

Supplementary Figure 8.

Peak pressure of antipodal ejecta sourced from the impactor as a function of their flight time.

Same as Fig. 3, but for the impact scenarios of 17.4 km/s.

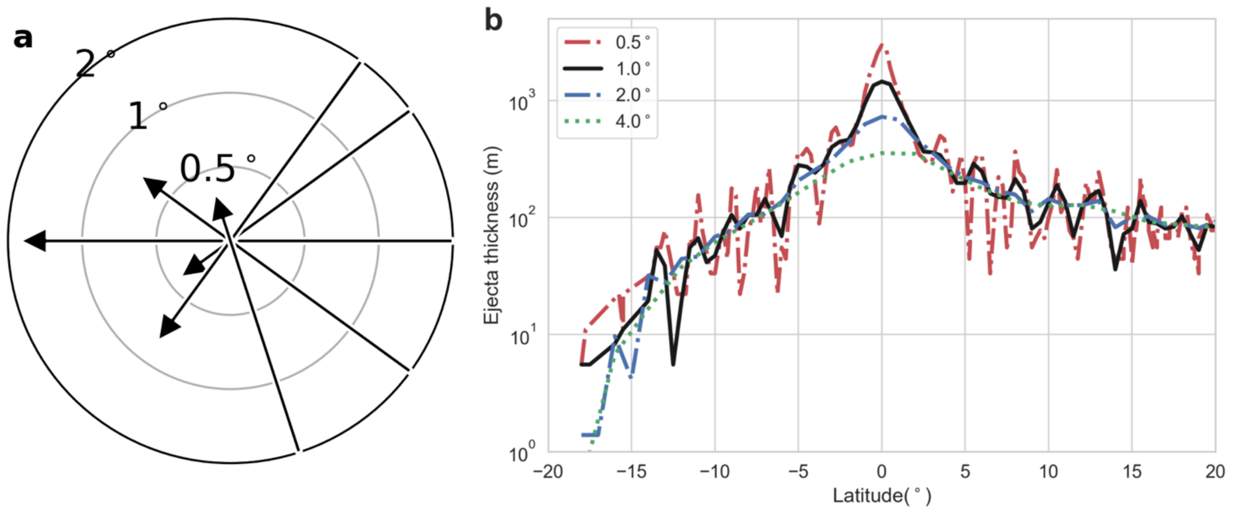

Supplementary Figure 9.

Thickness of ejecta around the antipode.

(a) Schematic image of trajectory of ejecta around the antipode. (b) Total ejecta thickness along the direction of 45º impactor at 12 km/s (see dotted line in Fig. 4 in the main text). 0º depicts the antipode, negative number denotes the direction over the antipode, and positive number denotes the opposite direction (between the antipode and the impact site). Each line represents a different sized area used to calculate the thickness (see legend).

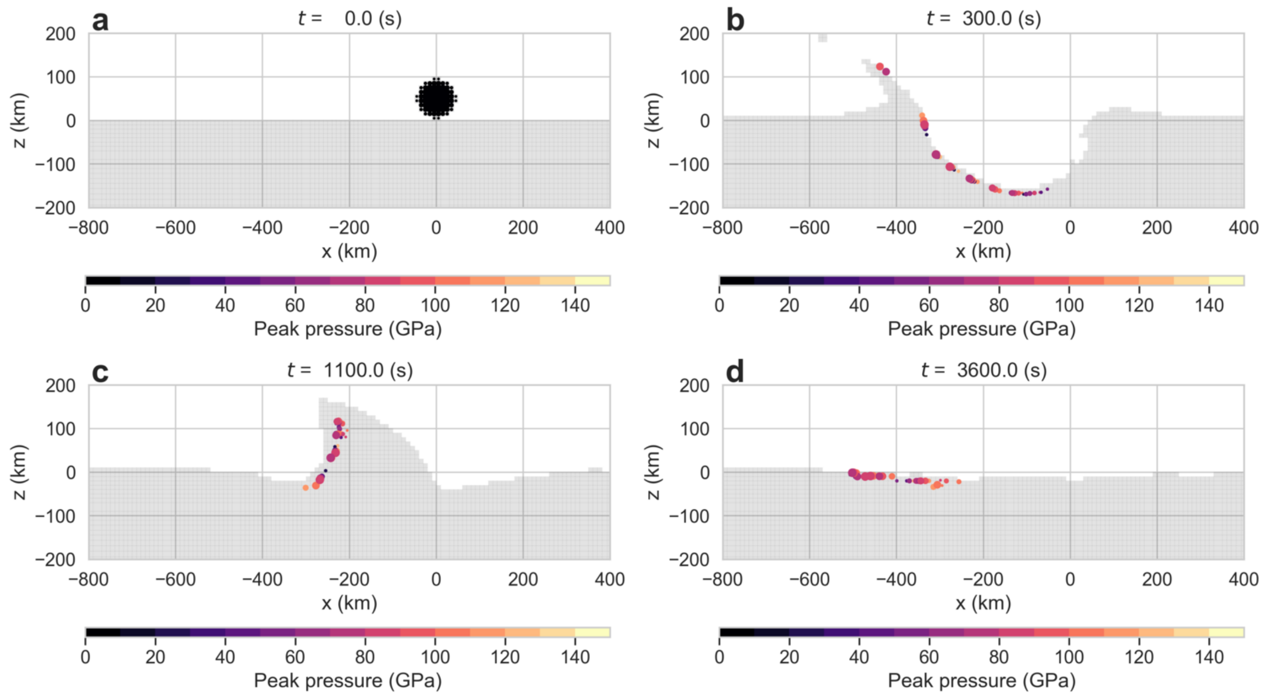

Supplementary Figure 10.

Time series of basin formation.

A 100-km-diameter impactor hits the target at (x, z) = (0, 0) at 12 km/s with 45° in direction from right to left. Each panel represents (a) t = 0 s, (b) t = 300 s, (c) t = 1100 (s), and (d) t = 3600 s, after the impact, respectively. Gray cells indicate the cross-sectional viewing at the impact site. Colored particles represent the peak pressure of the impactor material. Note that we only plot particles that are initially located on the symmetry (x-z) plane. The size of symbols indicates their original location in the impactor; the large ones are near the center and the smaller ones are near the surface.

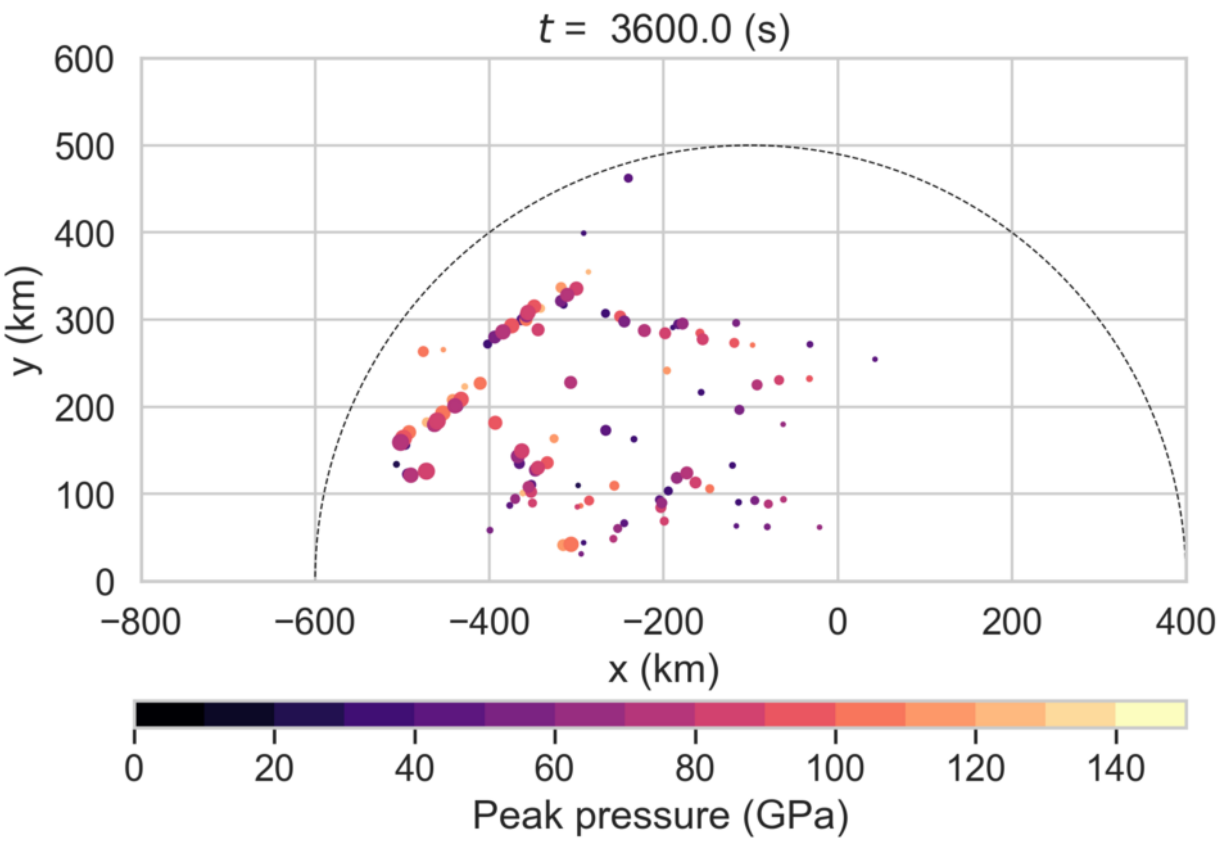

Supplementary Figure 11.

Ejecta distribution around the crater.

In contrast to Supplementary Fig. 10d, this illustrates top viewing. The viewing of colored particles is the same as in Supplementary Fig. 10. The dashed half circle illustrates a plausible location of a 1000 km-diameter-crater. Note that this is a tentative and the diameter would range 900 – 1100 km, due to the difficulty in recognizing the elevation.

Supplementary Table 1.

Antipodal thickness and its origin.

Impact angle	Impact velocity	Antipodal Thickness			Antipodal Thickness (unmelted)*		
		Total	Impactor	Target	Total	Impactor	Target
20º	12 km/s	856 m	100 m	756 m	856 m	100 m	756 m
	17.4 km/s	1117 m	94 m	1022 m	1086 m	72 m	1014 m
30º	12 km/s	2275 m	529 m	1746 m	2275 m	529 m	1746 m
	17.4 km/s	2025 m	429 m	1596 m	1408 m	194 m	1214 m
45º	12 km/s	1449 m	695 m	754 m	1238 m	557 m	682 m
	17.4 km/s	1194m	809 m	385 m	158 m	100 m	58 m
60º	12 km/s	0 m**	0 m**	NaN	0 m**	0 m**	NaN
	17.4 km/s	144 m	141 m	3 m	25 m	25 m	NaN

* We only consider unmelted ejecta and exclude melted ejecta.

** There are only two tracer particles (see main text).

Supplementary Table 2.

iSALE input parameters.

Description	Values
Equation of state	ANEOS
Bulk material of impactor/target	dunite [16]
Solidus temperature	1373 K [17]
Simon approximation constant A	1520 MPa [18]
Simon approximation exponent C	4.05 [18]
Poisson's ratio	0.25 [19]
Thermal softening parameter	1.1 [19]
Strength model	Rock [20]
Cohesion (damaged)	0.01 MPa [19]
Cohesion (undamaged)	10 MPa [19]
Frictional coefficient (damaged)	0.6 [19]
Frictional coefficient (undamaged)	1.2 [19]
Strength at infinite pressure	3.5 GPa [19]
Damage model	Ivanov [20]
Minimum failure strain	10^{-4} [19]
Damage model constant	10^{-11} [19]
Threshold pressure for damage model	300 MPa [19]
Number of high-resolution cells in x, y, z direction	700, 200, 250
Size of high-resolution cell	1 km

Supplementary References:

1. Tsunakawa, H. et al. Surface vector mapping of magnetic anomalies over the Moon using Kaguya and Lunar Prospector observations. *J. Geophys. Res.* **120**, 1160-1185 (2015).
2. Lawrence, D. J. et al. Iron abundances on the lunar surface as measured by the Lunar Prospector gamma-ray and neutron spectrometers. *J. Geophys. Res.* **107(E12)**, 5130 (2002).
3. Miljković, K. et al. Subsurface morphology and scaling of lunar impact basins. *J. Geophys. Res. Planets* **121**, 1695– 1712 (2016).
4. Johnson, B. C. et al. Spherule layers, crater scaling laws, and the population of ancient terrestrial impactors. *Icarus* **271**, 350-359, (2016).
5. Hood, L. L. & Artemieva, N. A. Antipodal effects of lunar basin-forming impacts: Initial 3D simulations and comparisons with observations. *Icarus* **193**, 485-502 (2008).
6. Davison, T. M., Collins, G.S., Elbeshausen, D., Wünnemann, K. & Kearsley, A. Numerical modeling of oblique hypervelocity impacts on strong ductile targets. *Meteoritics & Planetary Science*, **46**, 1510-1524. (2011).
7. Singer, K. N., Jolliff, B. L. & McKinnon, W. B. Lunar secondary craters and estimated ejecta block sizes reveal a scale-dependent fragmentation trend. *J. Geophys. Res. Planets* **125**, e2019JE006313 (2020).
8. Lin, R. P., Anderson, K. A. & Hood, L. L. Lunar surface magnetic field concentrations antipodal to young large impact basins. *Icarus* **74**, 529-541 (1988).
9. Mitchell D. L. et al. Global mapping of lunar crustal magnetic fields by Lunar Prospector. *Icarus* **194**, 401-409 (2008).
10. Baek, S.-M., Kim, K.-H., Garrick-Bethell, I. & Jin, H. Magnetic anomalies within the Crisium basin: Magnetization directions, source depths, and ages. *J. Geophy. Res. Planets* **124**, 223-242 (2019).

11. Johnson, B. C. et al. Formation of the Orientale lunar multiring basin. *Science* **354**, 441-444 (2016).

12. Wieczorek, M. A., Weiss, B. P. & Stewart, S. T. An Impactor Origin for Lunar Magnetic Anomalies. *Science* **335**, 1212-1215 (2012).

13. Dwyer, C. A., Stevenson, D. J. & Nimmo, F. A long-lived lunar dynamo driven by continuous mechanical stirring. *Nature* **479**, 212-214 (2011).

14. Parker, R. L. A theory of ideal bodies for seamount magnetism, *J. Geophys. Res.* **96(B10)**, 16101-16112 (1991).

15. Smith D. E. et al. Initial observations from the Lunar Orbiter Laser Altimeter (LOLA). Geophys. Res. Lett. 37, L18204 (2010).

16. Benz, W. Cameron, A. G. W. & Melosh, H. J. The origin of the Moon and the single-impact hypothesis III. *Icarus* **81**, 113-131 (1989).

17. Davison, T. M., Collins, G. S. & Bland, P. A. Mesoscale Modeling of Impact Compaction of Primitive Solar System Solids. *Astrophys. J.* **821**, 68 (2016).

18. Davison, T. M. "Numerical modelling of heat generation in porous planetesimal collisions" thesis, Imperial College London, London, U.K. (2010).

19. Kurosawa, K. & Genda, H. Effects of friction and plastic deformation in shock-comminuted damaged rocks on impact heating. *Geophys. Res. Lett.* **45**, 620– 626 (2018).

20. Collins, G. S., Melosh, H. J. & Ivanov, B. A. Modeling damage and deformation in impact simulations. *Meteorit. Planet. Sci.* **39**, 217–231 (2004).
